# Supplementary material for: Emergence of Weyl fermions by ferrimagnetism in a noncentrosymmetric magnetic Weyl semimetal
Source: Nat Commun. 2023 Nov 8;14:7185. doi: 10.1038/s41467-023-42996-8 (PMC10632385; doi:10.1038/s41467-023-42996-8)
Supplement: Supplementary file 1 — Supplementary Information [file 41467_2023_42996_MOESM1_ESM.pdf]

Supplementary Material for

Emergence of Weyl Fermions by Ferrimagnetism in a Noncentrosymmetric Magnetic Weyl Semimetal

Cong Li[1,2,♯,*], Jianfeng Zhang[3,♯], Yang Wang[1,♯], Hongxiong Liu[3,♯], Qinda Guo[1], Emile Rienks[4], Wanyu Chen[1], Francois Bertran[5], Huancheng Yang[6], Dibya Phuyal[1], Hanna Fedderwitz[7], Balasubramanian Thiagarajan[7], Maciej Dendzik[1], Magnus H. Berntsen[1], Youguo Shi[3], Tao Xiang[3], Oscar Tjernberg[1*]

[1]*Department of Applied Physics, KTH Royal Institute of Technology, Stockholm 11419, Sweden*

[2]*Department of Applied Physics, Stanford University, Stanford, CA 94305, USA*

[3]*Beijing National Laboratory for Condensed Matter Physics, Institute of Physics, Chinese Academy of Sciences, Beijing 100190, China*

[4]*Helmholtz-Zentrum Berlin für Materialien und Energie, Elektronenspeicherring BESSY II, Albert-Einstein-Straße 15, 12489 Berlin, Germany*

[5]*Synchrotron SOLEIL, L'Orme des Merisiers, Départementale 128, 91190 Saint-Aubin, France*

[6]*Department of Physics and Beijing Key Laboratory of Opto-electronic Functional Materials & Micro-nano Devices, Renmin University of China, Beijing 100872, China*

[7]*MAX IV Laboratory, Lund University, 22100 Lund, Sweden*

[♯]*These people contributed equally to the present work.*

[*]*Corresponding authors: conli@kth.se, oscar@kth.se*

1. Magnetic susceptibility measurements of NdAlSi

Figure S1a shows the magnetic field dependent isothermal magnetization measured at different temperatures. It can be clearly observed that when temperature at 2 K the magnetization M for H⊥c-axis (black line in Fig. S1a) is close to linear with field up to 9 T. But for H//c-axis it has two step-like transitions (the first transition: 0∼0.2 T; the second transition: 5.7 T∼6.1 T) and two stepped platforms. By normalizing magnetization to Bohr magneton, the atomic magnetic moment corresponding to the first platform is about $1\mu_B/Nd^{3+}$, while the atomic magnetic moment corresponding to the second platform is about $3\mu_B/Nd^{3+}$ (the full magnetization of free Nd^{3+} ion is $3.37\mu_B$[1]). Therefore, we can make a reasonable inference that the observed first platform (1/3 of the magnitude of full magnetization) originates from a ferrimagnetic state with an up-up-down (↑↑↓, UUD) [or a down-down-up (↓↓↑, DDU)] spin alignment. As the magnetic field continues to increase to about 6.1 T, the second transition platform appears corresponding to a spin flip transition which results in the transformation of the spin structure from the ferrimagnetic state with the UUD (or DDU) spin alignment to a ferromagnetic state with an up-up-up (↑↑↑, UUU) [or down-down-down (↓↓↓, DDD)] spin alignment. This picture is consistent with the recent neutron diffraction measurements[2]. In addition, as the temperature increases, the magnetization of this step-like transition is suppressed, and it tends to get linearized, as shown in Fig. S1a. Fig. S1b shows the temperature dependent susceptibility measured with different magnetic fields. At low magnetic fields (H<0.2 T), the susceptibility curve exhibits two transition points corresponding to the incommensurate (T_{inc}∼7.3 K) and commensurate (T_{com}∼3.4 K) ferrimagnetic state. When the applied magnetic field is in the first platform, the temperature dependent susceptibility curves exhibit the ferrimagnetic behavior with a Curie temperature at ∼7.3 K. While when the applied magnetic field is in the second transition range, the temperature dependent susceptibility curves show an antiferromagnetic behavior. As the applied magnetic field continues to increase to over ∼6.1 T, the susceptibility curve shows ferromagnetism (Fig. S1b). According to the above analysis, we find that the magnetic field may induce different magnetic order in NdAlSi at low temperature.

2. Calculated band structures of NdAlSi in UUD and DDU type ferrimagnetic state

Figure S2 shows the calculated band structures of NdAlSi along C-Γ-Z direction in UUD and DDU type ferrimagnetic state. It can be observed that the band structure calculations of UUD and DDU type ferrimagnets are basically coincident.

3. Calculated bulk Fermi surface of NdAlSi in the paramagnetic state

Figure S3a-S3c show the density functional theory (DFT) calculated three-dimensional (3D) bulk Fermi surface (Fig. S3a) and constant energy contours of NdAlSi at binding energy of 0.1 eV (Fig. S3b) and 0.2 eV (Fig. S3c) in the paramagnetic state. The bulk Fermi surface of NdAlSi in the paramagnetic state is composed of small independent Fermi pockets. Fig. S3d-S3f are the calcuated bulk Fermi surfac (Fig. S3d) and constant energy contours at binding energy of 0.1 eV (Fig. S3e) and 0.2 eV (Fig. S3f) of k_z=0 π/c plane which is helpful to quickly understand the bulk state electronic structure of NdAlSi.

4. Surface projected DFT band calculations of all possible cleavage surfaces in NdAlSi

According to the crystal structure of NdAlSi, it can be found that there are three cleavage possibilities along (001) direction, including the cleavage at the Nd-Al plane, at the Nd-Si plane and at the Al-Si plane. Therefore, six possible termination surfaces can be produced theoretically. In order to determine which kind of termination surface the experimental measurements come from, we did surface projection DFT band calculations along $\overline{M} - \overline{\Gamma} - \overline{Y} - \overline{M} - \overline{X} - \overline{\Gamma}$ directions on all possible termination surfaces, as shown in Fig. S4.

5. Domain structures in NdAlSi

After determinate the electronic structure measurements in this work is from Nd termination surfaces cleavage at the Al-Nd plane, we further performed the surface projected DFT Fermi surface and constant energy contours calculations in Fig. 2c. It can be found that the calculated Fermi surface exhibits two-fold symmetry, but the measured Fermi surface shows the four-fold symmetry. This leads us naturally to think that there may be two domain structures on the cleavage surface of NdAlSi. Through the crystal structure analysis can be found that the existence of two surface states from two orthogonal domains on the terminal surface of Nd cleavage plane of NdAlSi can also be understood from crystal

structure. There are two possible cleavage locations in NdAlSi at the Nd-Al plane (C1 and C2 in Fig. S5a), if the sample is cleaved from C1 in Fig. S5a, the distribution of Nd atoms on the fracture surface is shown in Fig. S5b (Domain1), while if the sample is cleaved from C2 in Fig. S5a, the distribution of Nd atoms on the fracture surface (Domain2, Fig. S5c) rotates 90 degrees with respect to Domain1 (Fig. S5b). The coexistence of the two orthogonal domains is consistent with the four-fold symmetry of the Fermi surface we measured (Fig. 2b and 2d), which the Fermi surface from one domain has two-fold symmetry (Fig. 2c).

6. Surface Fermi-arcs in NdAlSi

Fermi arcs in Weyl semimetals are a unique and important feature of the electronic structure. It is one part of the surface state that connects two Weyl nodes of opposite chirality in the bulk electronic structure. Therefore, different from the bulk state, surface Fermi arc does not exhibit dispersion along k_z direction. With the help of surface Green function calculations on the terminal surface of Nd atom cleavage at the Nd-Al plane, the Fermi arc can be visualized in Fig. S6a-S6b. Two Fermi arcs can be observed from it. One is at the energy of 38 meV above Fermi level (Fig. S6a), and the other one is at the energy of 56 meV above Fermi level (Fig. S6b). Fig. S6c-S6d show the calculated constant energy contours with considering two domain structures which derived from Fig. S6a-S6b and their superposition after rotation of 90 degrees. Fig. S7a-S7d show the photon energy dependent Fermi surface mapping. The arc features on Fermi surfaces exhibit negligible photon energy dependence, suggesting that they have surface state origin. The corresponding photon energy dependent band dispersion measurements of Fig. S7a-S7d along Cut1 direction are plotted in Fig. S7e-S7h. The second derivative plot of these band dispersions are show in Fig. S7i-S7l which exhibit the dispersion map of the Fermi arcs. A left-moving and right-moving edge mode related by a mirror plane k_x =0 can be observed clearly, corresponding to the Chern numbe n_l =-1 and n_r =+1, respectively.

7. Comparison of bulk Fermi surface and constant energy contours between experiments and calculations

In the measurement, we noted that vacuum ultraviolet (VUV) ARPES is a more sensitive surface measurement technique. In addition, NdAlSi has a strong surface state electronic structure. During the photoemission measurement, photoelectrons from surface states and

bulk states are mixed together, which makes it difficult to distinguish the electronic structure of the bulk states. In this case, people tend to use soft X-ray (SX)-ARPES, which is relatively sensitive to bulk states, for measurement. However, we found that in addition, the bulk state can also be protruded by making measurements on a low quality surface which the surface state can be suppressed. It is worth noting that low quality surface acquisition is a cleavage effect. NdAlSi is a three-dimensional non-centrosymmetric crystal, the single crystal after cleavage has a certain probability of showing a flat surface, a bad surface, and a surface in between. The corresponding location can be found by spatial scanning of the sample. Fig. S8a-S8f show the photon energy dependent Fermi surface (Fig. S8a) and constant energy contours at binding energy of 0.1 eV (Fig. S8b), 0.2 eV (Fig. S8c), 0.3 eV (Fig. S8d), 0.4 eV (Fig. S8e) and 0.5 eV (Fig. S8f). The Fermi surface measured at 41 eV on the low quality surface is completely different from the Fermi surface on a high quality surface (Fig. 2b). All features related to the surface states are basically missing from Fig. S8a. In order to understand the electronic structures measured in Fig. S8a-S8f, we did the DFT bulk Fermi surface calculations as shown in Fig. S8g-S8l. It can be found that the Fermi surface and constant energy contours measured with photon energy of 41 eV and 31 eV have a good agreement with the corresponding bulk Fermi surface and constant energy contours calculations of $k_z = 0\ \pi/c$ and $0.9\ \pi/c$ planes. In addition, the bulk calculations of $k_z = 0.2\ \pi/c$ and $0.4\ \pi/c$ planes can also capture most of the features of the measurements with photon energy of 38 eV and 45 eV. Therefore, we can confirm without any doubt that the electronic structures we measured on the low quality surface are mainly derived from the bulk electronic structures. In addition, we also performed SX-ARPES measurements on NdAlSi single crystal, as shown in Fig. S9, which can help us further understand the bulk states of NdAlSi.

8. Comparison of measurements between linear horizontal and positive circular polarization

Figure S10 shows the electronic structure measurements of NdAlSi with photon energy of 34 eV under linear horizontal (LH, Fig. S10a and S10c) and positive circular (PC, Fig. S10b and S10d) polarization. It can be found that the measurements under PC polarization show more detailed features relative to LH polarization. Therefore, in this work, we mainly use PC polarization for ARPES measurements.

9. Temperature dependent electronic structure measurements of NdAlSi

In this section, we show the temperature dependent electronic structure measurements of NdAlSi to further confirm that the observation of the extra features at 2 K are related to the UUD ferrimagnetic order. During the measurements, sample was cleaved at 15 K and immediately measured. Fig. S11a shows the measured constant energy contour at binding energy of 70 meV with photon energy of 41 eV at 15 K on a fresh surface. Fig. S11d is the corresponding band dispersion measurement along Cut1 in Fig. S11a at 15 K. After that, the sample was rapidly cooled down to 2 K and measured. The measured constant energy contour and the band dispersion along the same direction of Cut1 at 2 K are show in Fig. S11b and S11e. Then, the temperature of the sample was heated again to 15 K and similar measurements were made on it. It can be found that the features of the two measurements (Fig. S11a, S11d and Fig. S11c, S11f) at 15 K agree well with each other, except for the movement of the central electron band due to the adsorbed hole doping with time. But for the measurements at 2 K, an extra feature can be observed in Fig. S11b and S11e. To further visualized the band feature, we did MDCs analysis for the band dispersion (Fig. S11d-S11f), as shown in Fig. S11g-S11i. The green hollow dots in Fig. S11h mark the extra feature generated at 2 K, and it disappears at 15 K (Fig. S11g and S11i). Combined with the MDCs analysis, the extra feature at 2 K (marked by green arrow in Fig. S11e) is looks like a mirror-symmetric with the feature marked by red arrow in Fig. S11e. What is interesting is that the mirror axis of symmetry is on the Brillouin zone (BZ) boundary of the UUD ferrimagnetic state. Fig. S12 shows more detailed temperature dependent cut slices (Fig. S12c-S12d, Cut1-Cut5 in Fig. S12b) of band dispersion to exhibit the extra feature generated at 2 K (Fig. S12c). All the cut slices in Fig. S12c show the presence of extra features at 2 K (marked by green arrows in Fig. S12c) and these features are mirror-symmetric to the features marked by red arrows in Fig. S12c about the BZ boundary of the UUD ferrimagnetic state. Therefore, we can undoubtedly infer that the extra feature generated at 2 K is related to the ferrimagnetic order in NdAlSi.

[1] J. F. Wang *et al.*, NdAlSi: A magnetic Weyl semimetal candidate with rich magnetic phases and atypical transport properties. Phys. Rev. B **105**, 144435 (2022).

[2] J. Gaudet *et al.*, Weyl-mediated helical magnetism in NdAlSi. Nat. Mater. **20**, 1650 (2021).

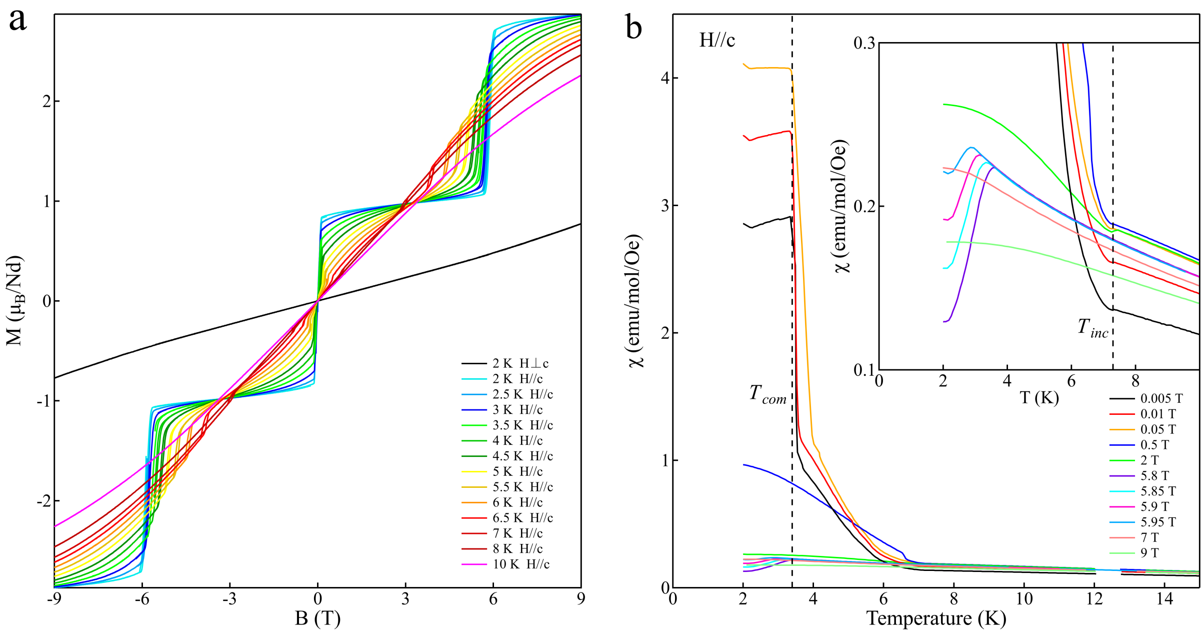

FIG. S1. **Magnetic susceptibility of NdAlSi.** (a) Magnetic field dependent isothermal magnetization M for H⊥c-axis (black line) and H//c-axis (color lines). (b) Temperature dependent magnetic susceptibility for H//c at magnetic fields from 0.005 T to 9 T. The inset in panel (b) is the zoom in view at temperatrure from 0 K to 10 K.

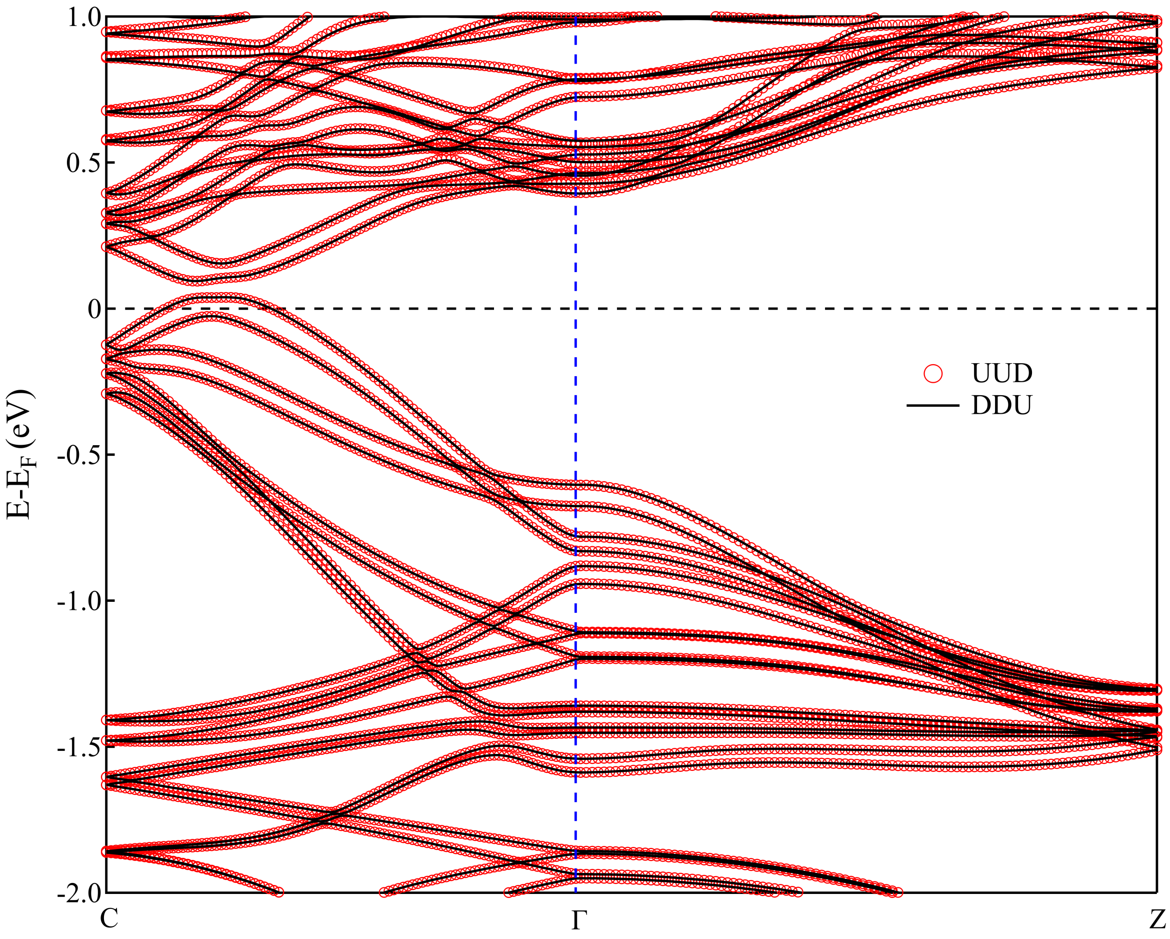

FIG. S2. **Comparetion of calculated band structures of NdAlSi along C-Γ-Z direction in UUD (red hollow dots) and DDU (black line) type ferrimagnetic state.**

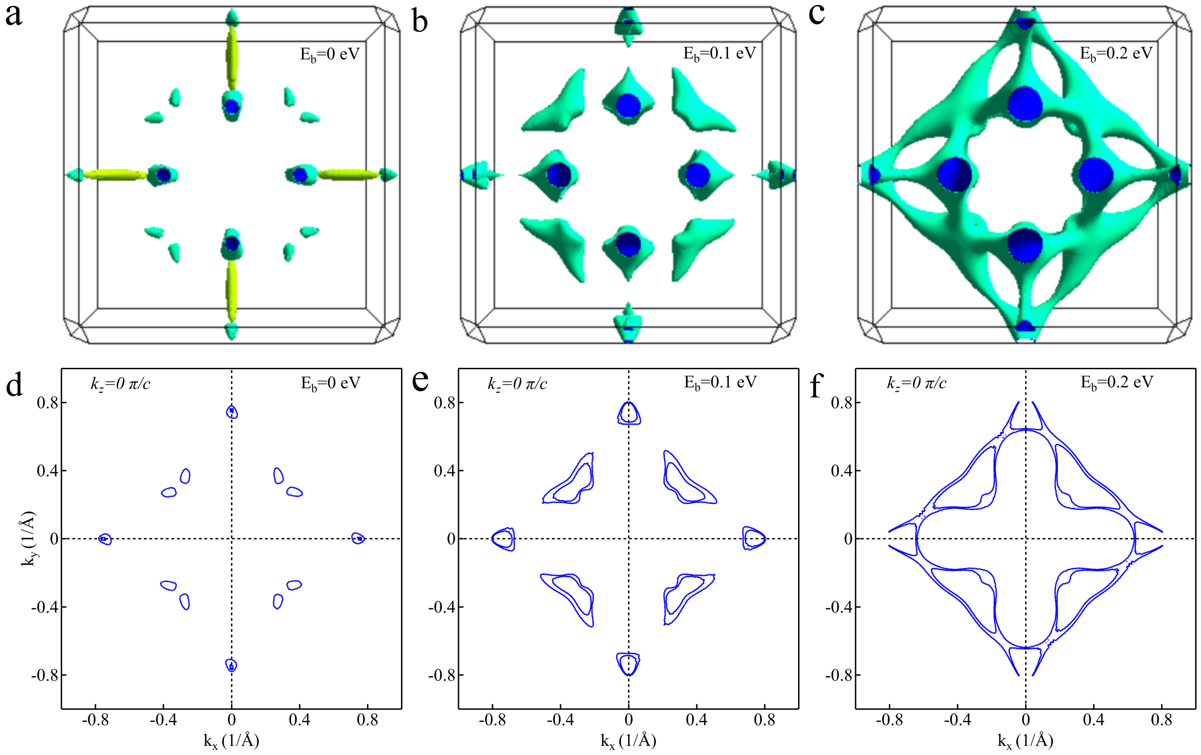

FIG. S3. **Calculated bulk Fermi surface of NdAlSi.** (a-c) The DFTcalculated 3D bulk Fermi surface and constant energy contours at different binding energies. (d-f) The DFTcalculated bulk Fermi surface and constant energy contours at different binding energies of k_z=0 π/c plane.

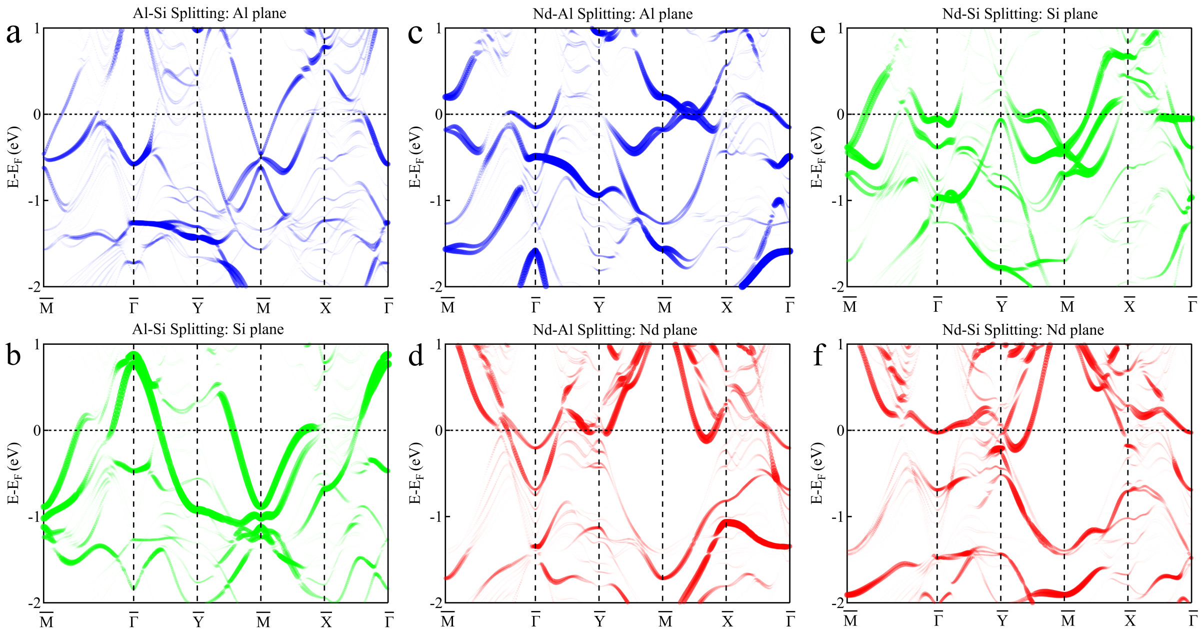

FIG. S4. **Surface projected DFT band calculations of NdAlSi.** (a-f) The surface projected DFT calculated band dispersion along $\overline{M}-\overline{\Gamma}-\overline{Y}-\overline{M}-\overline{X}-\overline{\Gamma}$ directions on the terminal surface of Al (a) and Si (b) atom cleavage at the Al-Si plane, Al (c) and Nd (d) atom cleavage at the Al-Nd plane, Si (e) and Nd (f) atom cleavage at the Nd-Si plane.

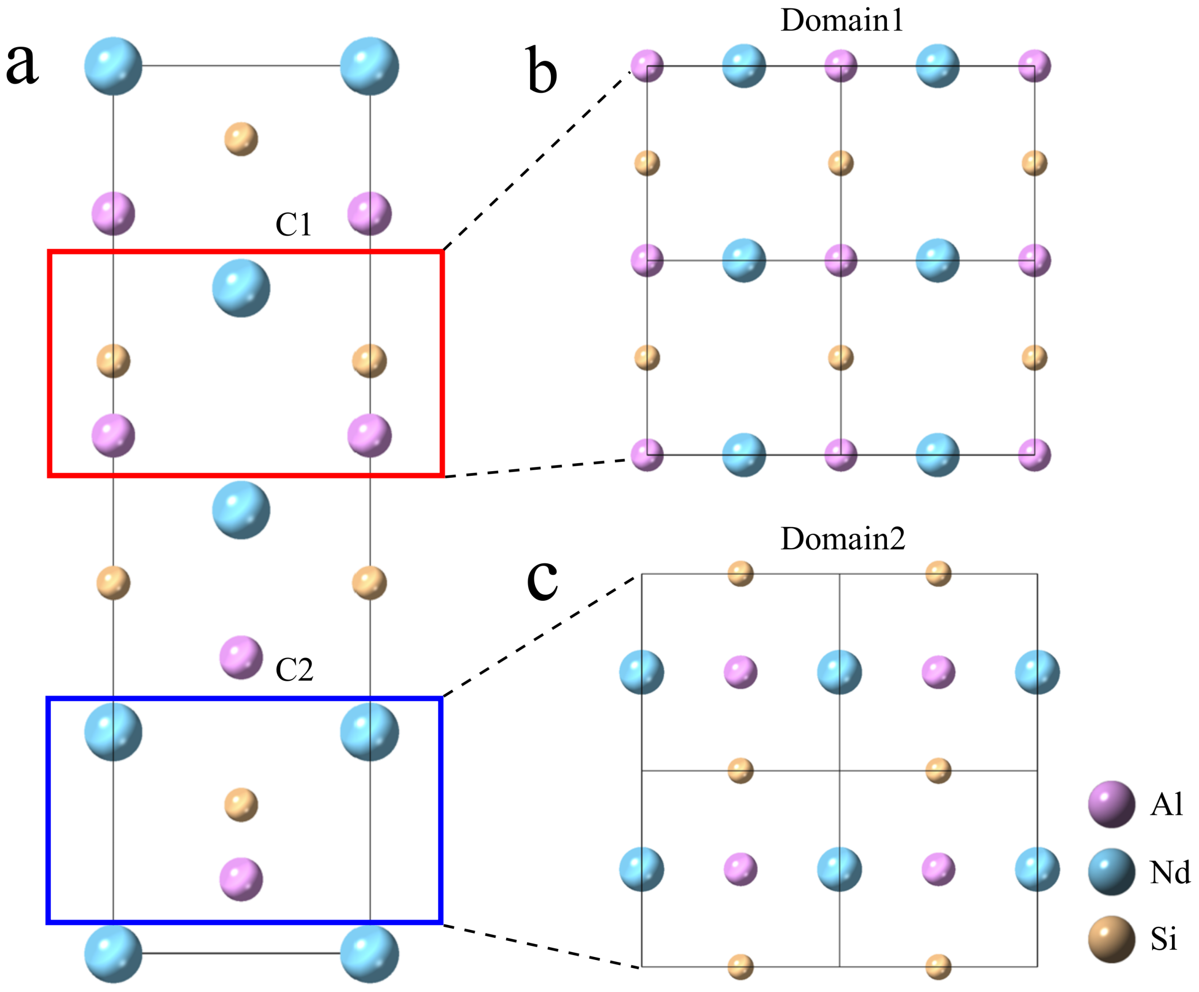

FIG. S5. **Lattice structure diagram of the cleavage plane of NdAlSi.** (a) The side view of the crystal structure of NdAlSi. (b-c) The top view of the cleavage plane from C1 (b) and C2 (c).

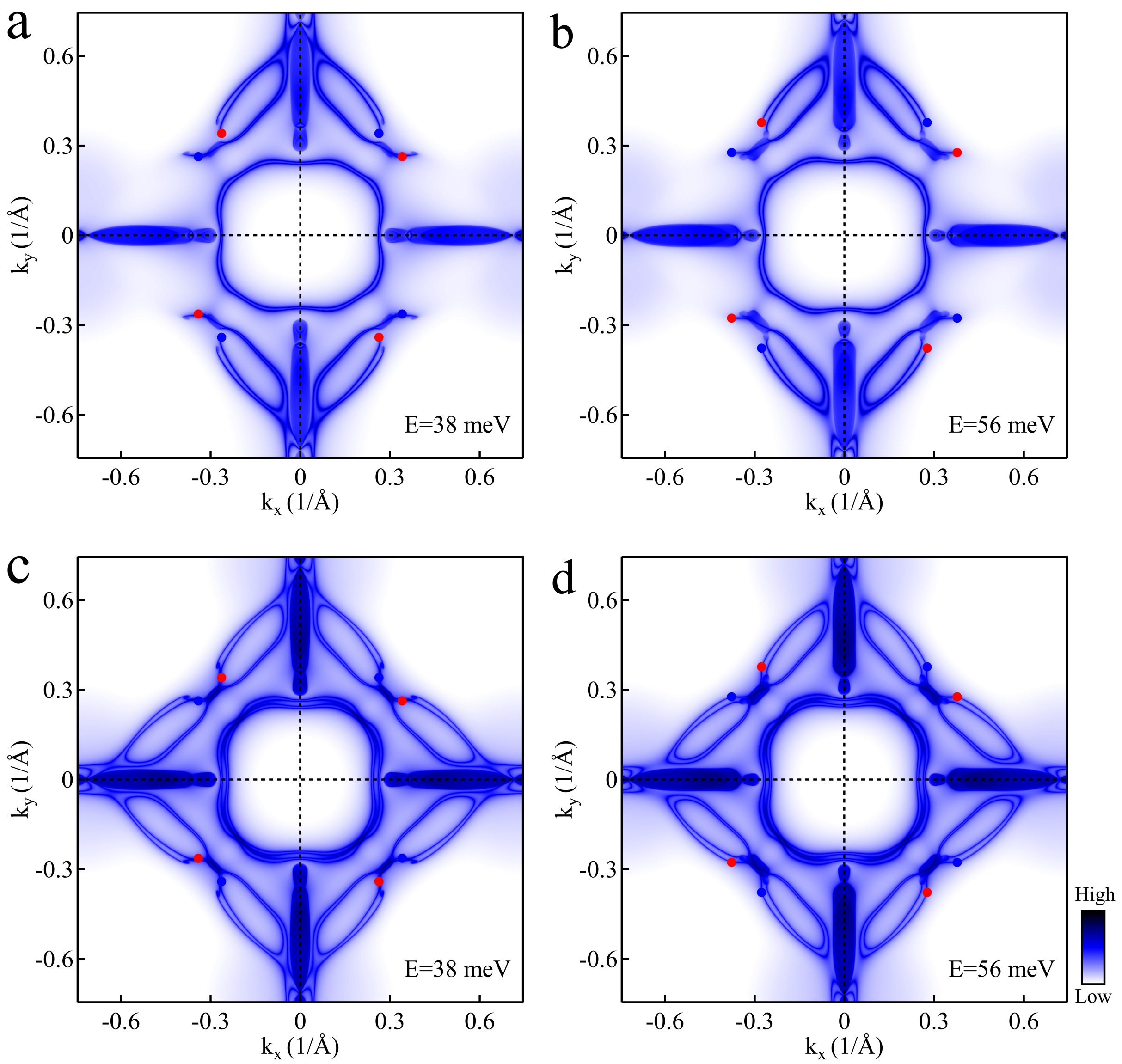

FIG. S6. **Calculated constant energy contours of NdAlSi on the terminal surface of Nd atom.** (a-b) Surface Green function calculation of constant energy contours at the energy of 38 meV (a) and 56 meV (b). (c-d) The calculated constant energy contours with considering two domain structures correspond to (a-b).

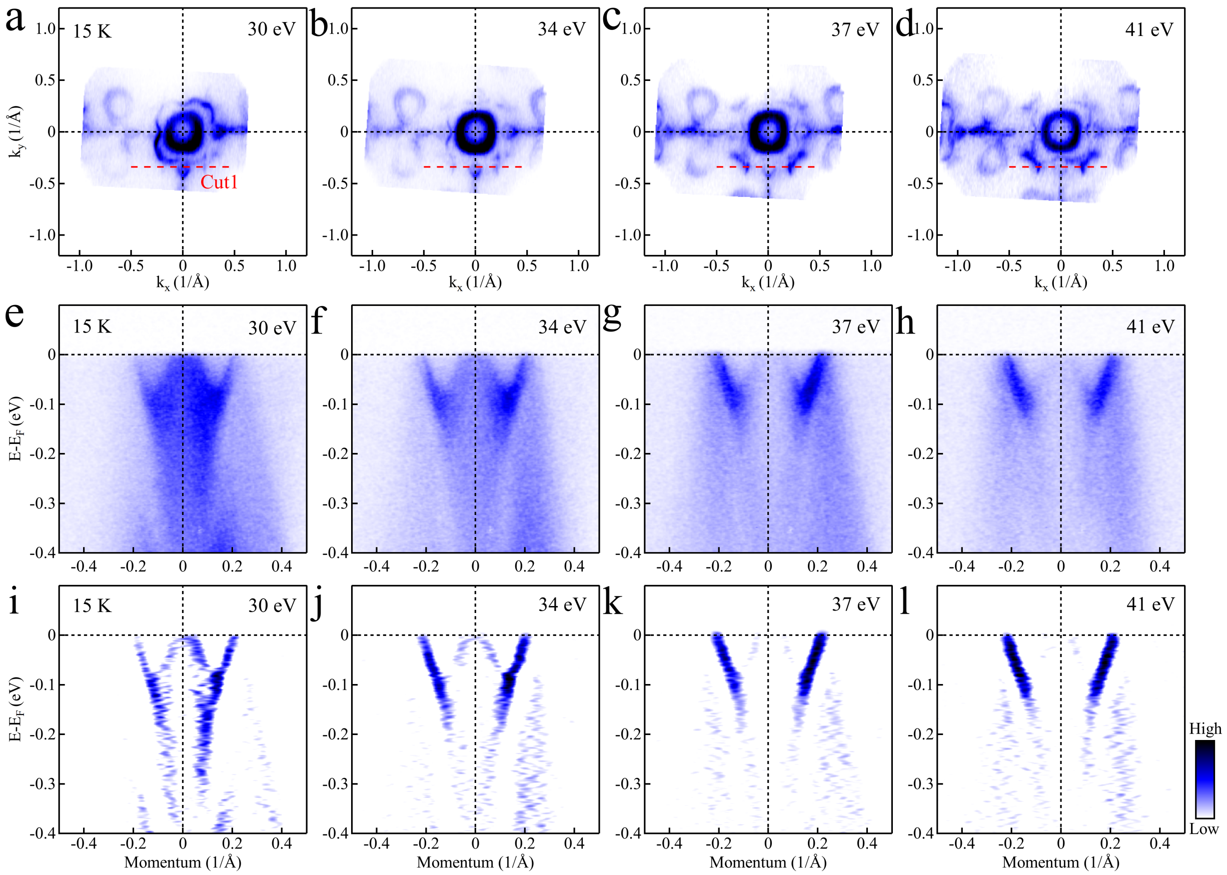

FIG. S7. **Photon energy dependent Fermi surface and band dispersion of NdAlSi.** (a) Fermi surface of NdAlSi measured at 15 K with the photon energy of 30 eV (a), 34 eV (b), 37 eV (c) and 41 eV (d) under positive circular (PC) polarization. (e-h) The photon energy dependent band dispersion measurements along Cut1-Cut4 directions at 15 K. The repeating figures of Fig. 2l-2o in the main text. (i-l) The corresponding momentum distribution curve (MDC) second derivative image of (e-h).

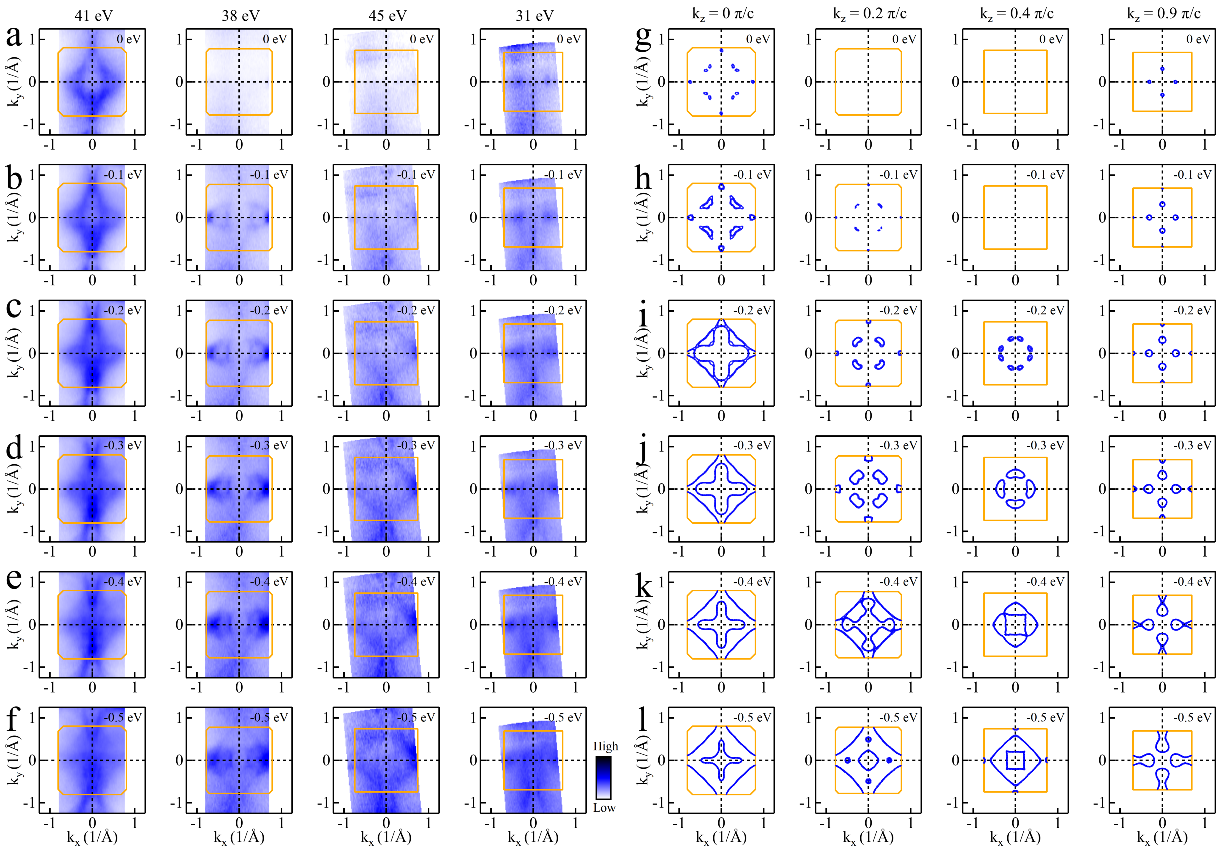

FIG. S8. **Comparison of measured and calculated Fermi surface and constant energy contours.** (a-f) The measured Fermi surface and constant energy contours at different binding energies with the photon energy of 41 eV, 38 eV, 45 eV and 31 eV. (g-l) The calculated Fermi surface and constant energy contours at different binding energies of k_z=0 π/c, 0.2 π/c, 0.4 π/c and 0.9 π/c planes.

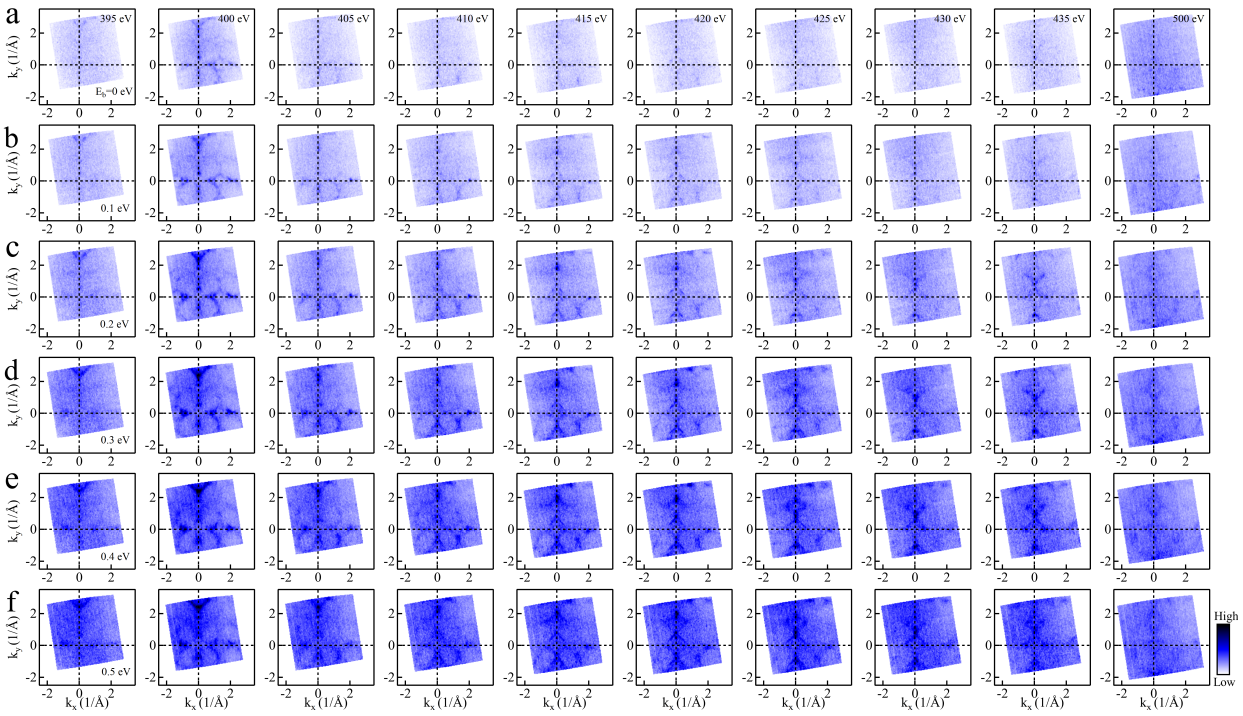

FIG. S9. **Fermi surface and constant energy contours of NdAlSi measured by SX-ARPES.** (a-f) Fermi surface and constant energy contours of NdAlSi at different binding energies measured with the photon energy of 395 eV, 400 eV, 405 eV, 410 eV, 415 eV, 420 eV, 425 eV, 430 eV, 435 eV and 500 eV.

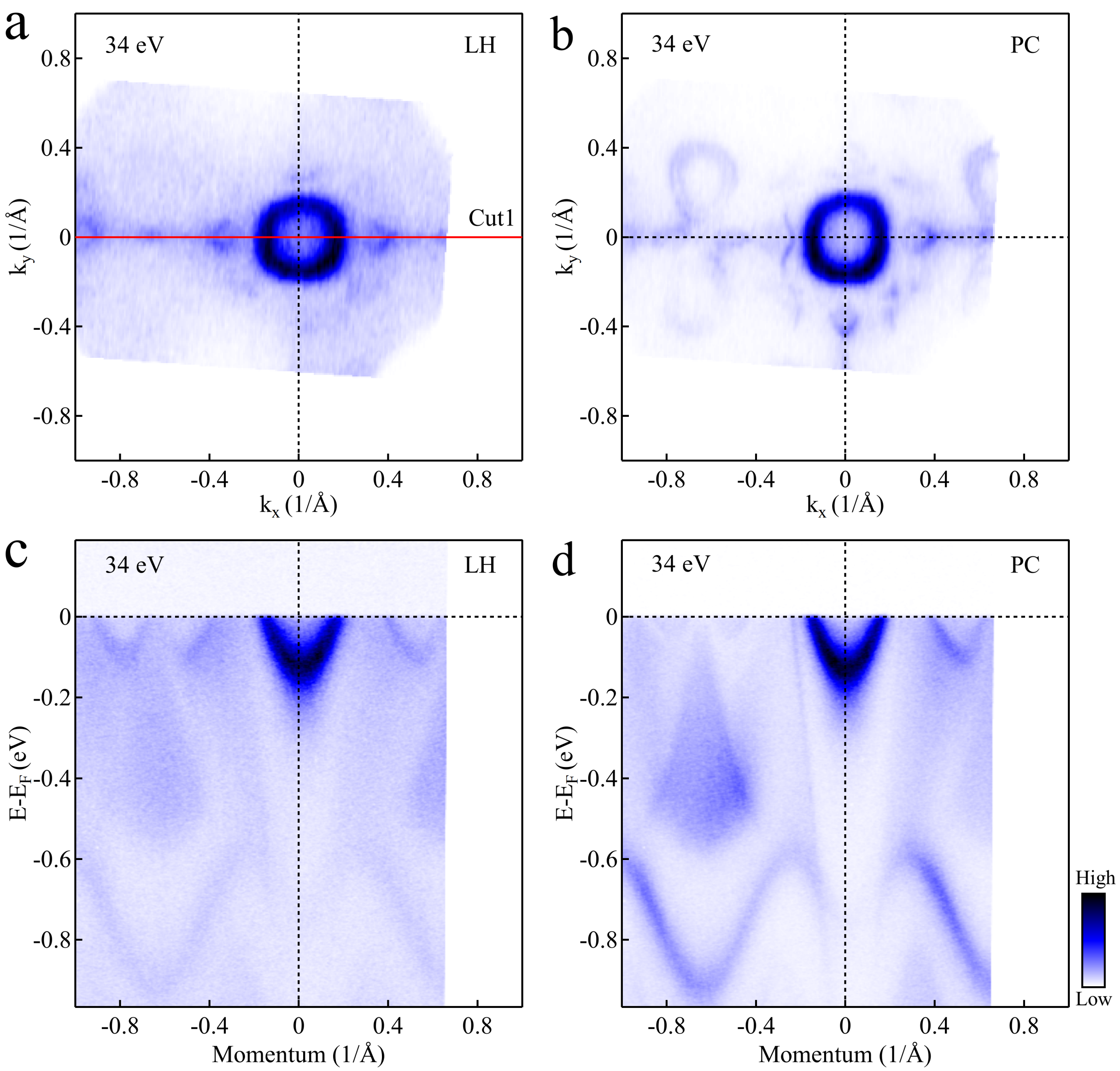

FIG. S10. **Comparison of the measurements with photon energy of 34 eV under LH and PC polarizations.** (a-b) The Fermi surface measured at 15 K with the photon energy of 34 eV under LH (a) and PC (b) polarizations. (c-d) The band dispersion measurements along Cut1 at 15 K with the photon energy of 34 eV under LH (c) and PC (d) polarizations.

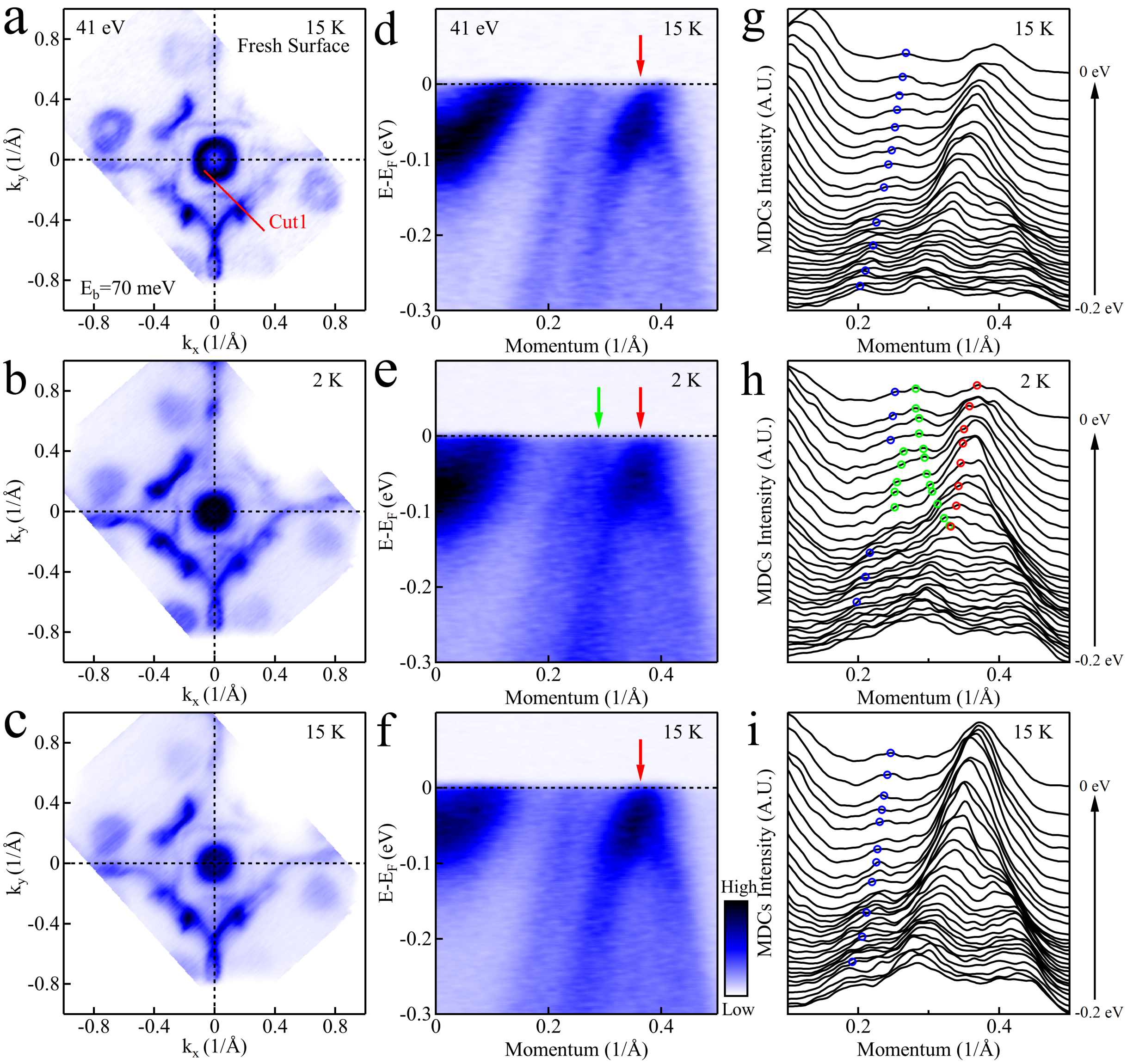

FIG. S11. **Temperature dependent electronic structure measurements of NdAlSi.** (a-c) The constant energy contours at binding energy of 70 meV measured with the photon energy of 41 eV at 15 K on an as cleaved surface (a), 2 K (b) and 15 K (c). (d-f) The corresponding band dispersion measurements of (a-c) along Cut1 direction in (a). (g-h) The corresponding MDCs analysis of (d-f).

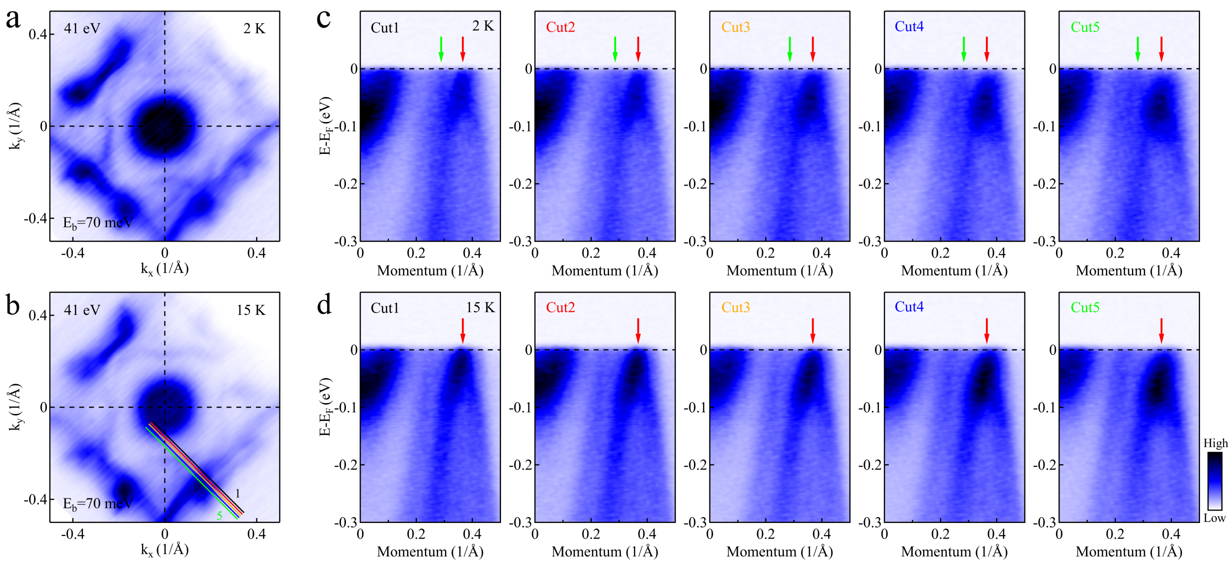

FIG. S12. **Comparison of temperature dependent cut slices of band dispersion.** (a-b) The constant energy contours at binding energy of 70 meV measured with the photon energy of 41 eV at 2 K (a) and 15 K (b). (c-d) The band dispersion measurements along Cut1-Cut5 directions at 2 K (c) and 15 K (d).
